# Insufficiency of prosthetic posterolateral overlap related to recurrence after laparoscopic transabdominal preperitoneal inguinal hernioplasty, as assessed by video review

**DOI:** 10.1186/s12893-020-0690-6

**Published:** 2020-02-10

**Authors:** Masanori Sato, Masashi Nozawa, Takahiro Watanabe, Takanobu Onoda, Atsuko Matsuyama, Norihiko Shiiya, Hidetoshi Wada

**Affiliations:** 1 First Department of Surgery, Hamamatsu University School of Medicine, 1-20-1 Handayama Higashiku, Hamamatsu, Shizuoka, 431-3192 Japan; 2 Department of Surgery, Shimada Municipal Hospital, 1200-5 Noda, Shimada, Shizuoka, Japan; 3 Department of Surgery, Yaizu City Hospital, 1000 Dobara, Yaizu, Shizuoka, Japan

**Keywords:** Hernia recurrence type, Laparoscopic hernioplasty, Retrospective case control study, Surgical techniques, TAPP, Video review

## Abstract

**Background:**

Technical factors leading to hernia recurrence after transabdominal preperitoneal repair include insufficient dissection, inadequate prosthetic overlap and prosthetic size, improper fixation and folding, or crinkling of the prosthesis. However, determining intraoperatively if a case will develop recurrent hernias due to these factors remains unclear.

**Methods:**

Five surgeons blind-reviewed operation videos of primary laparoscopic hernioplasty in 13 lesions that went on to develop recurrent hernias (i.e., future recurrence), as well as 28 control lesions, to assess twelve items of surgical techniques. Since we changed a surgical policy of covering myopectineal orifice (MPO) in April 2003, we analyzed the data for the earlier and later periods. The data was analyzed with hierarchical clustering to obtain a gross grouping. The differences of the ratings between the future recurrent and control lesions were then analyzed and the association of the techniques with the hernia recurrence rate, the size of the prosthesis, and the hernia type across hernia recurrence were explored.

**Results:**

The lesions were grouped based on the time series, and its boundary was approximated when we changed our surgical policy. This policy change caused ratings to progress from 34% satisfactory, to 79% satisfactory. The recurrence rate decreased to 0.7% (5/678), compared with 6.2% (10/161) before the policy was implemented (*p* < 0.001). With univariate analysis, the ratings of posterior prosthesis overlap to the MPO in the recurrent lesions were significantly lower than controls in the later period (*p* = 0.019). Although various types of recurrences were noted in the earlier period, only primary indirect and recurrent indirect hernias were observed in the later period (*p* = 0.006).

**Conclusions:**

Fully covering the MPO with mesh is essential for preventing direct recurrence hernias. Additional hernia recurrence prevention can be obtained by giving appropriate attention to prosthesis overlap posterior to the MPO in a large indirect hernia.

## Background

The number of laparoscopic hernioplasty (LH) cases for groin hernia in Japan has been increasing since the medical fee was revised under the National Health Insurance System in 2012. During the 13th nationwide survey of endoscopic surgery in 2015, LH accounted for 41.3% of all groin hernia repair surgeries [1]. However, unacceptable recurrence rates of 3.0% with the transabdominal preperitoneal (TAPP) approach and 3.4% with the total extraperitoneal approach were reported in the 2014 and 2015 surveys, possibly due to an uptick in the number of inexperienced surgeons performing the surgery for the first time.

Technical factors leading to hernia recurrence include insufficient dissection, inadequate prosthetic overlap, insufficient prosthetic size, improper fixation, folding or crinkling of a prosthesis, and missed hernias [2]. These factors were found responsible for nearly all cases of recurrence, and covering Fruchaud’s myopectineal orifice (MPO) entirely by a prosthetic mesh with adequate overlap was reported to be essential for preventing hernia recurrence [3]. Indeed, the use of a large mesh sheet decreased hernia recurrence [4, 5]. These techniques should be assessed during surgery to determine whether the lesion will develop a recurrent hernia (i.e., future recurrence). However, which technical factors support the prediction of future recurrence remains unclear.

The purpose of this study was to identify a surgical technique that predicts future hernia recurrence in TAPP repair and to clarify the mechanisms associated with the technique. For this, we conducted a video review assessment of future recurrent and control lesions. A multiple-evaluation method was employed by plural reviewers to increase the integrity and objectivity of the evaluation. Surgical techniques (such as adequate dissection, mesh coverage, and folding or crinkling of the mesh) were assessed at the time of primary repair while accounting for the type of hernia in primary and recurrent lesions, according to the Japan Hernia Society (JHS; see Additional file 1: Table S1) classification [6].

## Methods

### Future recurrent lesions and video materials to be reviewed

Between April 1992 and July 2017, 839 lesions were repaired in 711 patients using TAPP at Hamamatsu University School of Medicine. Our criteria and the operative procedure used to perform TAPP have been described previously [7]. Fifteen patients subsequently suffered recurrence after we performed TAPP repair (Table 1). Recurrent cases were based on voluntary visits by the patient or referral visits from a cooperating doctor. Thirteen videos of the primary LH were used for review in this study; of the remaining two videos, one was missing, and one did not have a second operation performed. Eleven videos of the reoperations and the two written operation records were used to assess the types of hernia recurrence. Two or three controls per case were selected from among the individuals who were matched according to categorical variables (such as patient gender, type of hernia, operation date, and surgeon). The control patients were examined or interviewed to confirm that there had been no hernia recurrence.

**Table 1:** Data for primary or secondary surgery of the all recurrent cases

Case	Age	Sex	Year of primary LH	History of ipsilateral hernia repair (times)	Duration before recurrence (months)	Surgeon experience of the case	Primary LH video availability	Laterality	JHS classification of primary hernia	JHS classification of recurrent hernia	Size of hernia orifice (mm)	Size of prosthesis (cm)	Secondary operation
23R	50’s	M	1994	0	64	22*	Yes	Rt.	II-3	II-3	23	11 × 6	TAPP
40	70’s	M	1995	1	2	3	Yes	Lt.	I-2	I-2	13	9 × 6	TAPP
55	60’s	M	1996	2	12	1†	Yes	Rt.	I-2	II-1	10	10 × 7	TAPP
56	50’s	M	1996	4	100	1	Yes	Lt.	II-1	II-2	7	11 × 8	TAPP
68	60’s	M	1997	1	6	39*	Yes	Lt.	II	II	NA	11 × 7	Ant.
95	50’s	M	1999	0	19	14	Yes	Rt.	I-2	NA	20	10 × 7	ND
102	70’s	M	2000	0	2	10	Yes	Rt.	II-1	II	NA	7 × 7	Ant.
103	60’s	M	2000	0	34	3	No	Lt.	II	II-1	NA	10 × 8	TAPP
106	70’s	M	2000	0	38	10	Yes	Rt.	II-3	I-2	30	11 × 8	TAPP
113	70’s	M	2000	0	7	3	Yes	Lt.	II-3	I-2	20	9 × 8	TAPP
167	70’s	M	2004	1	7	15	Yes	Rt.	I-2	I-2	20	12 × 10	TAPP
239	60’s	M	2006	0	18	34†	Yes	Rt.	I-2	I-2	25	14 × 9	TAPP
259	30’s	M	2007	0	18	44†	Yes	Rt.	I-3	I-3	30	14 × 9	TAPP
262	70’s	M	2007	0	13	4	Yes	Rt.	I-3	I-2	30	15 × 9	TAPP
528	50’s	M	2013	0	7	73	Yes	Rt.	I-3	I-3	35	15 × 10	TAPP

*, † indicates the same surgeon

*LH* laparoscopic hernioplasty; *NA* not available; *Ant* Anterior approach; *ND* not done.

### Video review assessments

**Fig. 1:**
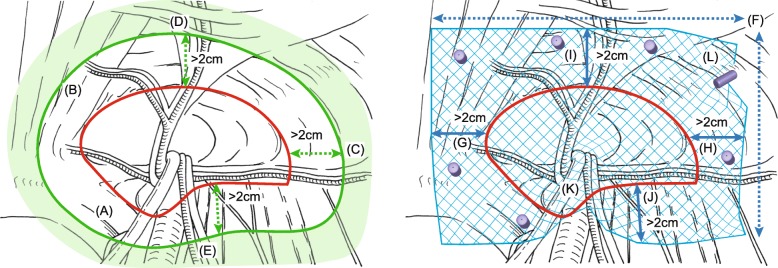
Assessment items for video review. Left panel, items regarding dissection (green zone): (**a**) dissection until exposure of Cooper’s ligament, (**b**) dissection until exposure of the rectus abdominis muscle, (**c**) dissection > 2 cm lateral from the lateral triangle, (**d**) dissection > 2 cm anterior from the hernia orifice, (**e**) dissection > 2 cm posterior to the hernia orifice. Right panel, items regarding mesh size or placement (blue mesh): (**f**) sufficient prosthesis size, (**g**) prosthesis overlap > 2 cm medial to the MPO, (**h**) prosthesis overlap > 2 cm lateral to the MPO, (**i**) prosthesis overlap > 2 cm anterior to the MPO, (**j**) prosthesis overlap > 2 cm posterior to the MPO, (**k**) presence of mesh folding or crinkling posteriorly, and (**l**) prosthesis fixation (purple button). Red lines show the MPO

Five surgeons, aside from MS, reviewed these approximately 15-min video digests of the primary LHs, that were anonymized and blinded for relapse, to assess the twelve items regarding applied surgical techniques (Fig. 1). The distance of the overlaps was referenced in previous reports [5, 8]. The reviewers assessed each item as either satisfactory (1 point), unsatisfactory (0 points), or undeterminable (uncounted). The average point of the countable choices was regarded as the rating for each item. A rating of 1.0 meant all reviewers assessed the item as satisfactory, whereas a rating of 0.0 meant all assessed it as unsatisfactory. The HemI software program (The CUCKOO Workgroup, China) was used to heat map the data, and perform hierarchical clustering using average linkage and the Manhattan distance similarity metric [9].

### Surgical techniques to predict hernia recurrence, recurrence rate and type of recurrence hernia

Since our surgical policy was changed to require covering the entire MPO with more than 2 cm overlap of the prosthesis in April 2003, we set the earlier period (April 1992 to March 2003) and the later period (April 2003 to July 2017). The differences of the ratings in each assessment item were assessed between future recurrences and controls for the two periods separately. Recurrence rates and prosthesis sizes were calculated using the hernia database of Hamamatsu University School of Medicine. MS reviewed the videos of recurrent hernia repair to determine the type of recurrent hernia and the location of the prosthesis. For cases repaired with an anterior approach, the written operation records were used to assess the type of recurrence. Recurrence rates, prosthesis sizes and type of recurrent hernia were examined by comparing between the earlier and later periods.

### Statistical analyses

The internal consistency among the reviewers was calculated using IBM SPSS software (IBM Corp., Armonk, NY, USA). When undeterminable responses were included in a question, the question was excluded for the reliability statistics. The Fisher–Freeman–Halton exact test was performed using the EZR software program (Jichi Medical University Saitama Medical Center, Japan) [10]. The other statistical analyses were performed using IBM SPSS software. With the nonparametric Mann–Whitney U test, the null hypothesis was “the distribution of ratings for the two groups is the same”. A *p*-value of < 0.05 was considered to be statistically significant.

## Results

We reviewed the primary LH videos of 13 future recurrence and 28 control lesions. The basic characteristics, including the surgeon’s degree of experience, were not significantly different between the groups (Table 2). The 12-item questionnaire completed for each of the 41 videos demonstrated good internal consistency among the reviewers (Cronbach’s α co-efficient = 0.816).

**Table 2:** Characteristics of future recurrent and control lesions used for the video reviews

	Future recurrent (*n* = 13)	Control (*n* = 28)	*p*-value
Gender, male:female	13:0	28:0	
Age (years)	63.8 ± 11.8	62.5 ± 15.0	0.847*
Operation time (min)	117 (65–158)	122 (60–324)	0.294†
Laterality, Lt.: Rt.	5:8	10:18	1.000^¶^
JHS type, I: II	7:6	16:12	1.000^¶^
JHS type, Primary: Recurrence	10:3	26:2	0.304^¶^
Surgeon’s experience (cases)	12.5 (1–72)	10.0 (1–73)	0.897†

*Student’s t-test, †Mann-Whitney U Test, ^¶^ Fisher’s exact test

*JHS* Japan Hernia Society.

### Division into two periods: before and after the policy change

**Fig. 2:**
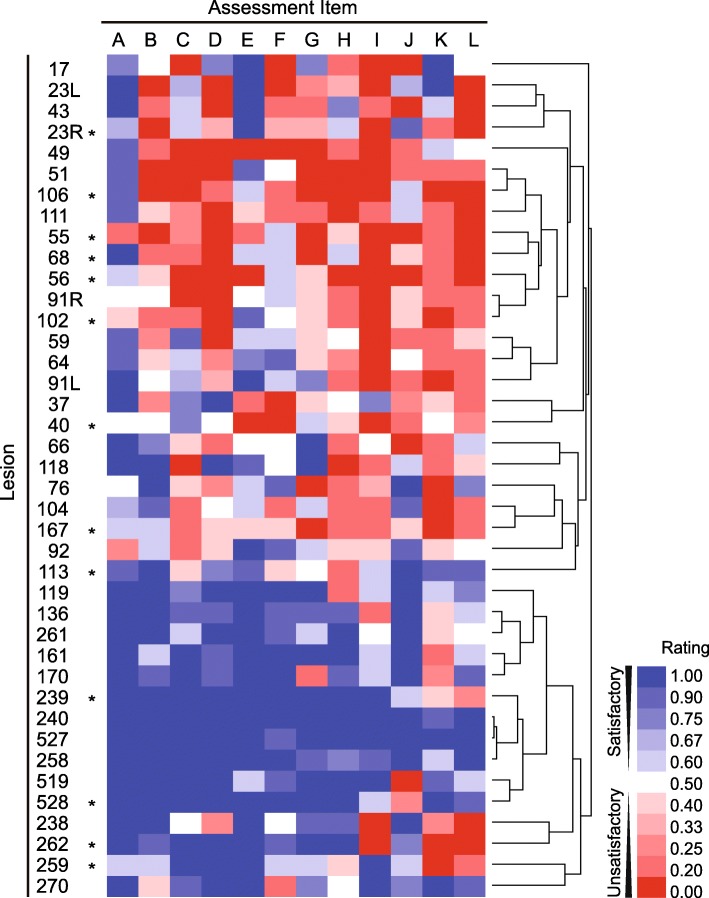
Heatmap and hierarchical clustering of video review assessment ratings. A cell shows an average rating of the video review assessment among the five reviewers with the colors, where satisfactory ratings are shown in blue and unsatisfactory ones in red. The relation of the rating and color is shown in the color bar panel. The serial numbers of the lesions are shown on the left-hand vertical axis, and the lesions that suffered hernia recurrence are marked with asterisks. The clustering tree is shown on the right-hand vertical axis

To determine how entire lesions were grouped according to their rating differences, the lesions were rearranged using hierarchical clustering (Fig. 2). There were two main branches on the y-axis. The upper branch comprised lesions 17 to 118, except for lesion 167, while the lower branch comprised lesions 136 to 528, except for lesion 119. This grouping was mostly divided by the period of operation, yet was not based on whether the lesions were future recurrent or not. Our surgical policy change occurred between lesions 136 and 161, it was close to a boundary of the above grouping between lesions 118 and 136. We therefore subsequently analyzed the data for the two periods separately. In the earlier period, 175 (58%) of the 300 questions were rated less than or equal to 0.4, indicating unsatisfactory ratings were predominant. Conversely, 143 (79%) of the 180 questions were rated greater than or equal to 0.6 during the later period, indicating generally satisfactory ratings and that the policy looked adhered to thoroughly.

### Surgical techniques intraoperatively predict hernia recurrence after TAPP repair

**Fig. 3:**
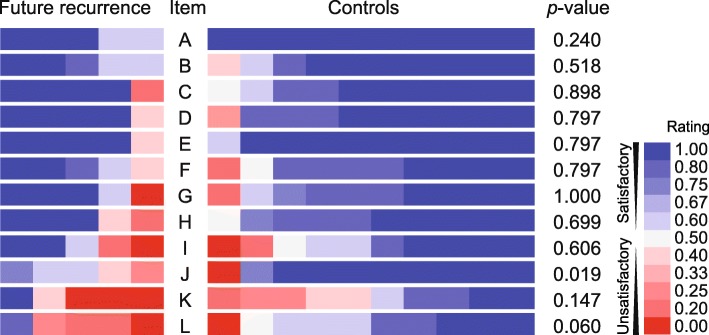
Colored rating table of video review assessment in the later period. Colored cells show video review assessment ratings for each lesion, sorted according to rating value in each item. The relation of the rating and color is shown in the color bar panel, where satisfactory ratings are shown in blue and unsatisfactory ones in red. Univariate analysis of the items for future recurrence and control lesions in the later period was completed using the Mann–Whitney U test, with *p* values shown on the right

To determine which techniques could predict hernia recurrence after TAPP repair, we assessed the differences of the ratings in each item between future recurrences and controls. In the earlier period, no items were significantly different between the future recurrences and controls in a univariate analysis (Additional file 1: Table S2). As unsatisfactory ratings were predominant in the controls, no techniques could predict future recurrence in the period. In the later period, we rearranged the heat map table according to rating values in order to make the differences between future recurrences and controls in each item conspicuous (Fig. 3). Regarding the ratings of the posterior prosthesis overlap (item J), three of the five recurrent lesions were rated 0.4 to 0.6, whereas eight of the 10 control lesions were rated 1.0, with a significant difference in a univariate analysis (*p* = 0.019). The probability of recurrence was higher when the reviewers agreed the posterior overlap was insufficient.

Regarding the ratings of prosthesis folding or crinkling posteriorly (item K), there was no significant difference between the two groups. However, unsatisfactory ratings of 0.0 were observed in three of the five recurrent lesions and, conversely, in none of the controls. The strong agreement of an unsatisfactory rating on the item among the reviewers, indicating an apparent technical error. Regarding the ratings of prosthesis fixation (item L), four of the five future recurrent lesions were rated less than 0.40, compared to only one of the 10 controls receiving a similar rating. The ratings in the future recurrences tended to be lower than the controls, although the difference was insignificant (*p* = 0.060).

### Differences in recurrence rates and their forms before and after the policy change

To appreciate how such surgical technique errors affected hernia recurrence, we investigated the recurrence rates for each period and each type of recurrent hernia. Due to general observations of unsatisfactory ratings during the earlier period and satisfactory ratings during the later period, these ratings were regarded as examples of all TAPP repairs for each period. When we inspected the recurrence rates for the two periods, the recurrence rate dropped drastically from 6.2% (10/161) during the earlier period to 0.7% (5/678) in the later period (chi-squared test, *p* < 0.001). A gross satisfactory rating in the later period should affected to enlargement of the prosthesis size. The average size of the prosthesis in the later period was 13.3 by 9.4 cm, while the earlier period’s average was 9.9 by 7.2 cm, indicating a significantly difference (Student’s t-test, *p* < 0.001). This indicates that fully covering the MPO resulted in the usage of a larger prosthesis, which helped reduce the recurrence rate.

We explored the differences in the types of hernia recurrence between the two periods. Both Type I and II recurrences were observed in the earlier period, where six of the nine cases relapsed with Type II, while three relapsed with Type I (Table 3). There were three cases in which the hernia types changed from Type I to II (or Type II to I) when the hernias relapsed. In contrast, the five cases that relapsed in the later period were all primary and recurrent Type I (*p* < 0.006). Furthermore, when reviewing the reoperation videos in the five observed later period cases, the meshes shifted anteriorly and/or medially to the hernia orifices, but not posteriorly. The sizes of the orifices were more than 30 mm in the three most recent cases, which were all classified as JHS Type I-3. Consequently, fully covering the MPO effectively reduced Type II recurrence. Even when fully covering the MPO, insufficient posterior overlap of the prosthesis would lead to Type I hernia recurrence.

**Table 3:** Type of recurrence according to the date of primary LH

JHS Classification		Date of the primary LH		*p* value*
Primary LH	Recurrence	In the earlier period (*n* = 9) ^a^	In the latter period (*n* = 5)	
II	II	5	0	0.006
I	II	1	0	
II	I	2	0	
I	I	1	5	

^a^ Case 103 was added to the data, *Fisher-Freeman-Halton exact test

*LH* laparoscopic hernioplasty, *JHS* Japan Hernia Society.

## Discussion

Previous analyses of surgical techniques leading to recurrence were based on reviews of primary LH videos, findings on reoperation, or comparative studies [2, 5, 8, 11]. As distinguishing the conditions that lead to recurrence during primary surgery is important, we compared the primary LH videos of future recurrence and control lesions in a blinded fashion. Given that an evaluation by a single inspector might be viewed as subjective despite abundant experience, we employed a multiple evaluation method to increase its integrity and objectivity. This multiple evaluation method was feasible because the internal consistency was found to be good among the five reviewers.

Time series was the dominant factor in the hierarchical clustering analysis. We set the two time periods that divided procedures when our institution adopted a policy to fully cover the MPO with more than 2 cm overlap. In fact, sufficiently covering the MPO in the later period was reasoned, by the general satisfactory rating of the assessment and larger size of the prosthesis. This resulted in a drastic reduction in the recurrence rate from 6.2 to 0.7% across the policy implementation. Leibl et al. (5) reported a recurrence rate of 2.8% (when using a 13 by 8 cm mesh with a slit) decreased to 0.36% when using a 15 by 10 cm mesh without a slit. Kapiris et al. (4) similarly found a 5% recurrence rate with an 11 by 6 cm mesh decreased to 0.16% with a 15 by 10 cm mesh. The mesh sizes in our study, even in the later period, tended to be smaller than 15 by 10 cm, but this fact may be due to the physical characteristics of the Japanese population. Fully covering the MPO resulted in the usage of a larger prosthesis, which helped reduce the rate of hernia recurrence. This supports the mesh size being the most important factor in preventing recurrence [12].

In the later period, 79% of the choices were assessed as satisfactory. As there were no cases of Type II recurrence during the later period, fully covering the MPO appears to be directly involved in preventing Type II recurrence. However, the fact that the five most recent recurrent cases experienced Type I recurrence with meshes located anterior and medial to the orifice highlights the existence of some particular mechanism that causes such recurrence. An insufficient overlap width of less than 2 cm posterior to the MPO is considered to play a role in the mechanism of Type I hernia recurrence, as indicated by the unsatisfactory ratings in the recurrent lesions. Due to the contraindication of tacking in this area, securing the overlap width and increasing its friction resistance are crucial in preventing recurrence [13]. Therefore, we have made an effort to secure an overlap of ≥3 cm from the edge of the MPO in all directions from 2009. Nonetheless, a larger posterior overlap is required for large hernias, as seen in the last three lesions, which had relatively larger orifices. The International Endohernia Society guidelines also recommend the use of a larger mesh (i.e., 12 by 17 cm or greater) for large hernia defects [14]. Since concrete data regarding the hernia defect size have yet to be obtained, the distance inferior to the iliopubic tract that the overlap should be secured for a large indirect hernia remains an issue.

In this study, we assessed the presence (but not the degree) of folding or crinkling of the prosthesis. The fact that unsatisfactory ratings of 0.0 for item K were observed only in the recurrent lesions indicated that prosthesis folding or crinkling was obvious to any reviewer given such lesions. As the assessment of prosthesis fixation is appreciably subjective, there were conflicting ratings among the reviewers in the four controls for item L, yet the reviewers agreed it to be unsatisfactory in four of the five future recurrences. These suggest that items K and L remain important factors in causing hernia recurrence when these technical errors are apparent.

Surgeon experience is also an important factors in hernia recurrence [15]. The number of experienced cases before an observed case was quite low for the future recurrent cases (Table 1). Since we employed the single hand technique with the assistance of an experienced surgeon in most cases, it was difficult to evaluate surgeon experience. Of the six surgeons undergoing archived learning curve analysis in our institution, half of them showed a learning curve in which 20–30 cases were necessary to enhance their surgical skills, but the half did not show any learning curves. To reduce the influence of surgeon experience, we selected controls that were matched according to categorical variables, including operation date and surgeon. As a result, the number of the surgeon experience cases was not significantly different between future recurrences and controls (Table 2). Together with inexperienced hands, we assessed which technical factors were responsible for hernia recurrence.

## Conclusion

Covering the MPO with mesh during TAPP repair made it possible to effectively reduce the frequency of hernia recurrence, especially for Type II recurrence. However, even when fully covering the MPO, a prosthesis overlap that occurred posterior to the MPO was found to be the most important factor leading to hernia recurrence, especially in large Type I hernias.

## Supplementary information

**Additional file 1: Table S1**. The type of groin hernia excerpted from The Japan Hernia Society (JHS) Classification. **Table S2**. Results of a univariate analysis of the items for future recurrence and control cases in the earlier period.

## Data Availability

The datasets used and/or analysed during the current study available from the corresponding author on reasonable request.

## Abbreviations

JHS: Japan Hernia Society
LH: Laparoscopic hernioplasty
MPO: Myopectineal orifice
TAPP: Transabdominal preperitoneal

## References

[1] Shiroshita H, Inomata M, Bandoh T, Uchida H, Akira S, Hashizume M, Yamaguchi S, Eguchi S, Wada N, Takiguchi S (2019). Endoscopic surgery in Japan: The 13th national survey (2014–2015) by the Japan Society for Endoscopic Surgery. Asian J Endosc Surg.

[2] Lowham AS, Filipi CJ, Fitzgibbons RJ, Stoppa R, Wantz GE, Felix EL, Crafton WB (1997). Mechanisms of hernia recurrence after preperitoneal mesh repair. Traditional and laparoscopic. Ann Surg.

[3] Fitzgibbons RJ, Camps J, Cornet DA, Nguyen NX, Litke BS, Annibali R, Salerno GM (1995). Laparoscopic inguinal herniorrhaphy. Results of a multicenter trial. Ann Surg.

[4] Kapiris SA, Brough WA, Royston CM, O'Boyle C, Sedman PC (2001). Laparoscopic transabdominal preperitoneal (TAPP) hernia repair. A 7-year two-center experience in 3017patients. Surg Endosc.

[5] Leibl BJ, Schmedt CG, Kraft K, Ulrich M, Bittner R (2000). Recurrence after endoscopic transperitoneal hernia repair (TAPP): causes, reparative techniques, and results of the reoperation. J Am Coll Surg.

[6] Japanese hernia society (2009). Japanese hernia society classification of inguinal hernia.

[7] Wada H, Kimura T, Kawabe A, Sato M, Miyaki Y, Tochikubo J, Inamori K, Shiiya N (2012). Laparoscopic transabdominal preperitoneal inguinal hernia repair using needlescopic instruments: a 15-year, single-center experience in 317 patients. Surg Endosc.

[8] Phillips EH, Rosenthal R, Fallas M, Carroll B, Arregui M, Corbitt J, Fitzgibbons RJ, Seid A, Schultz L, Toy F (1995). Reasons for early recurrence following laparoscopic hernioplasty. Surg Endosc.

[9] Deng W, Wang Y, Liu Z, Cheng H, Xue Y (2014). HemI: a toolkit for illustrating heatmaps. PLoS One.

[10] Kanda Y (2013). Investigation of the freely available easy-to-use software 'EZR' for medical statistics. Bone Marrow Transplant.

[11] Felix E, Scott S, Crafton B, Geis P, Duncan T, Sewell R, McKernan B (1998). Causes of recurrence after laparoscopic hernioplasty. A multicenter study. Surg Endosc.

[12] Neumayer L, Giobbie-Hurder A, Jonasson O, Fitzgibbons RJ, Dunlop D, Gibbs J, Reda D, Henderson W (2004). Open mesh versus laparoscopic mesh repair of inguinal hernia. N Engl J Med.

[13] Hollinsky C, Gobl S (1999). Bursting strength evaluation after different types of mesh fixation in laparoscopic herniorrhaphy. Surg Endosc.

[14] Bittner R, Arregui ME, Bisgaard T, Dudai M, Ferzli GS, Fitzgibbons RJ, Fortelny RH, Klinge U, Kockerling F, Kuhry E (2011). Guidelines for laparoscopic (TAPP) and endoscopic (TEP) treatment of inguinal hernia [International Endohernia Society (IEHS)]. Surg Endosc.

[15] Kukleta JF (2006). Causes of recurrence in laparoscopic inguinal hernia repair. J Minim Access Surg.

