# Supplementary material for: Insufficiency of prosthetic posterolateral overlap related to recurrence after laparoscopic transabdominal preperitoneal inguinal hernioplasty, as assessed by video review
Source: BMC Surg. 2020 Feb 10;20:27. doi: 10.1186/s12893-020-0690-6 (PMC7011534; doi:10.1186/s12893-020-0690-6)
Supplement: Supplementary file 1 — Additional file 1: Table S1. The type of groin hernia excerpted from The Japan Hernia Society (JHS) Classification. Table S2. Results of a univariate analysis of the items for future recurrence and control cases in the earlier period. [file 12893_2020_690_MOESM1_ESM.docx]

**Supplemental Table 1.** The type of groin hernia excerpted from The Japan Hernia Society (JHS) Classification. Originally published on the web at <http://jhs.mas-sys.com/classification.html>.

**Type I. Indirect (lateral) inguinal hernia**

**I-1. Indirect (lateral) inguinal hernia (small):** The diameter of the orifice defines less than 1cm (1 fingerbreadth); provided that less than 1 fingerbreadth defines that the fifth finger cannot be inserted into.

**I-2. Indirect (lateral) inguinal hernia (medium):** The diameter of the orifice defines equal or larger than 1cm (1 fingerbreadth) and less than 3cm (2 fingerbreadths); provided that less than 2 fingerbreadths define that the second and third finger cannot be inserted into.

**I-3. Indirect (lateral) inguinal hernia (large):** The diameter of the internal ring defines equal or larger than 3cm (2 fingerbreadths).

**Type II. Direct (medial) inguinal hernia**

**II-1. Direct (medial) inguinal hernia (supravesical):** The diameter of the orifice defines less than 3cm (2 fingerbreadths), and the center of the orifice is within the medial half of the posterior wall of the inguinal canal.

**II-2. Direct (medial) inguinal hernia (localized type):** The diameter of the orifice defines less than 3cm (2 fingerbreadths), and the center of the orifice is within the lateral half of the posterior wall of the inguinal canal.

**II-3. Direct (medial) inguinal hernia (diffuse type):** The diameter of the orifice defines equal or larger than 3cm (2 fingerbreadths).

**Type III. Femoral hernia**

**Type IV. Combined type**

Coexisting of indirect (lateral) inguinal hernia, direct (medial) inguinal hernia, or femoral hernia (describe each type)

**Type V. Unclassified with the upper types**

**Recurrent hernia** is described according to the classification of the primary hernia.

**Supplemental Table 2. Results of a univariate analysis of the items for future recurrence and control cases in the earlier period**

| Item | Future recurrence  (median)  (n=8) | Controls  (median)  (n=17) | *p*-value* |
| --- | --- | --- | --- |
| A | 0.63 | 0.80 | 0.086 |
| B | 0.20 | 0.50 | 0.124 |
| C | 0.23 | 0.40 | 0.628 |
| D | 0.10 | 0.25 | 0.588 |
| E | 0.60 | 0.75 | 0.288 |
| F | 0.45 | 0.50 | 0.842 |
| G | 0.37 | 0.40 | 0.238 |
| H | 0.30 | 0.20 | 0.669 |
| I | 0.00 | 0.20 | 0.157 |
| J | 0.40 | 0.40 | 0.932 |
| K | 0.20 | 0.20 | 0.511 |
| L | 0.00 | 0.20 | 0.097 |

*Mann–Whitney U test

MPO, myopectineal orifice
